# Sphingosine Kinase-2 Maintains Viral Latency and Survival for KSHV-Infected Endothelial Cells

**DOI:** 10.1371/journal.pone.0102314

**Published:** 2014-07-10

**Authors:** Lu Dai, Karlie Plaisance-Bonstaff, Christina Voelkel-Johnson, Charles D. Smith, Besim Ogretmen, Zhiqiang Qin, Chris Parsons

**Affiliations:** 1 Research Center for Translational Medicine and Key Laboratory of Arrhythmias of the Ministry of Education of China, East Hospital, Tongji University School of Medicine, Shanghai, China; 2 Department of Medicine, Louisiana State University Health Sciences Center, HIV Malignancies Program, Louisiana Cancer Research Center, New Orleans, Louisiana, United States of America; 3 Department of Microbiology/Immunology/Parasitology, Louisiana State University Health Sciences Center, HIV Malignancies Program, Louisiana Cancer Research Center, New Orleans, Louisiana, United States of America; 4 Departments of Microbiology/Immunology, Hollings Cancer Center, Medical University of South Carolina, Charleston, South Carolina, United States of America; 5 Department of Drug Discovery/Biomedical Sciences, Hollings Cancer Center, Medical University of South Carolina, Charleston, South Carolina, United States of America; 6 Department of Biochemistry/Molecular Biology, Hollings Cancer Center, Medical University of South Carolina, Charleston, South Carolina, United States of America; University of Southern California Keck School of Medicine, United States of America

## Abstract

Phosphorylation of sphingosine by sphingosine kinases (SphK1 and SphK2) generates sphingosine-1-phosphate (S1P), a bioactive sphingolipid which promotes cancer cell survival and tumor progression *in vivo*. We have recently reported that targeting SphK2 induces apoptosis for human primary effusion lymphoma (PEL) cell lines infected by the Kaposi’s sarcoma-associated herpesvirus (KSHV), and this occurs in part through inhibition of canonical NF-κB activation. In contrast, pharmacologic inhibition of SphK2 has minimal impact for uninfected B-cell lines or circulating human B cells from healthy donors. Therefore, we designed additional studies employing primary human endothelial cells to explore mechanisms responsible for the selective death observed for KSHV-infected cells during SphK2 targeting. Using RNA interference and a clinically relevant pharmacologic approach, we have found that targeting SphK2 induces apoptosis selectively for KSHV-infected endothelial cells through induction of viral lytic gene expression. Moreover, this effect occurs through repression of KSHV-microRNAs regulating viral latency and signal transduction, including miR-K12-1 which targets IκBα to facilitate activation of NF-κB, and ectopic expression of miR-K12-1 restores NF-κB activation and viability for KSHV-infected endothelial cells during SphK2 inhibition. These data illuminate a novel survival mechanism and potential therapeutic target for KSHV-infected endothelial cells: SphK2-associated maintenance of viral latency.

## Introduction

Kaposi’s sarcoma–associated herpesvirus (KSHV) is one of the most common etiologic agents for cancers, including primary effusion lymphoma (PEL) and Kaposi’s sarcoma (KS), arising preferentially in the setting of HIV infection [1], [2] or organ transplantation [3], [4]. Despite the reduced incidence of KS during antiretroviral therapy (ART), KS remains the most common AIDS-associated tumor worldwide [5] and continues to incur significant morbidity and mortality for these patients [1], [2]. Endothelial cell-derived, KSHV-infected “spindle cells” represent the defining histological feature of KS [6]. However, there are no therapeutic approaches available for selective targeting of these or other KSHV-infected cells within the tumor microenvironment. A better understanding of mechanisms regulating survival selectively for KSHV-infected cells relative to uninfected cells should facilitate development of safer and more effective therapeutic approaches for KSHV-infected tumors.

Sphingolipid biosynthesis involves hydrolysis of ceramides to generate sphingosine which is subsequently phosphorylated by one of two sphingosine kinase isoforms (SphK1 or SphK2) to generate sphingosine-1-phosphate (S1P) [7]–[10]. Bioactive sphingolipids, including ceramides and S1P, act as signaling molecules to regulate apoptosis and tumor cell survival [7]. In contrast to the generally pro-apoptotic function of ceramides, S1P promotes cell proliferation and survival [8]. Relatively limited published data also suggest a role for sphingolipid biosynthesis pathways in viral pathogenesis [11]–[13]. This is of particular relevance given the recent development of a highly selective and well-characterized small molecule inhibitor of SphK2 (ABC294640) [14], [15] displaying significant anti-tumor activity for a variety of cancers [16], [17] and which is currently under evaluation in a Phase I clinical trial for patients with solid tumors (Clinicaltrials.gov Identifier: NCT01488513). However, mechanistic and functional consequences of SphK inhibition for virus-infected cells had not been previously explored. We recently reported that pharmacologic inhibition of SphK2 using ABC294640 induces dose-dependent, caspase-mediated apoptosis for KSHV-infected PEL cell lines and suppresses PEL tumor progression *in vivo* [18]. Furthermore, we found that targeting SphK2 induces PEL apoptosis in part through inhibition of NF-κB activation. In contrast, pharmacologic inhibition of SphK2 had minimal to no impact on basal levels of bioactive sphingolipids, caspase cleavage, or cell survival for an uninfected B-cell tumor line, or for circulating human B cells from healthy donors [18]. These latter data suggest that SphK2 may selectively maintain survival for KSHV-infected cells, although additional work using relevant cell culture systems permissive for *de novo* KSHV infection are needed for exploration of potential mechanisms. Therefore, we sought to establish whether targeting SphK2 induces apoptosis selectively for KSHV-infected primary human endothelial cells following *de novo* infection, and if so, to identify putative mechanisms for SphK2-mediated survival of these cells.

## Results

### Targeting SphK2 induces caspase cleavage and apoptosis selectively for KSHV-infected endothelial cells

**Figure 1:**
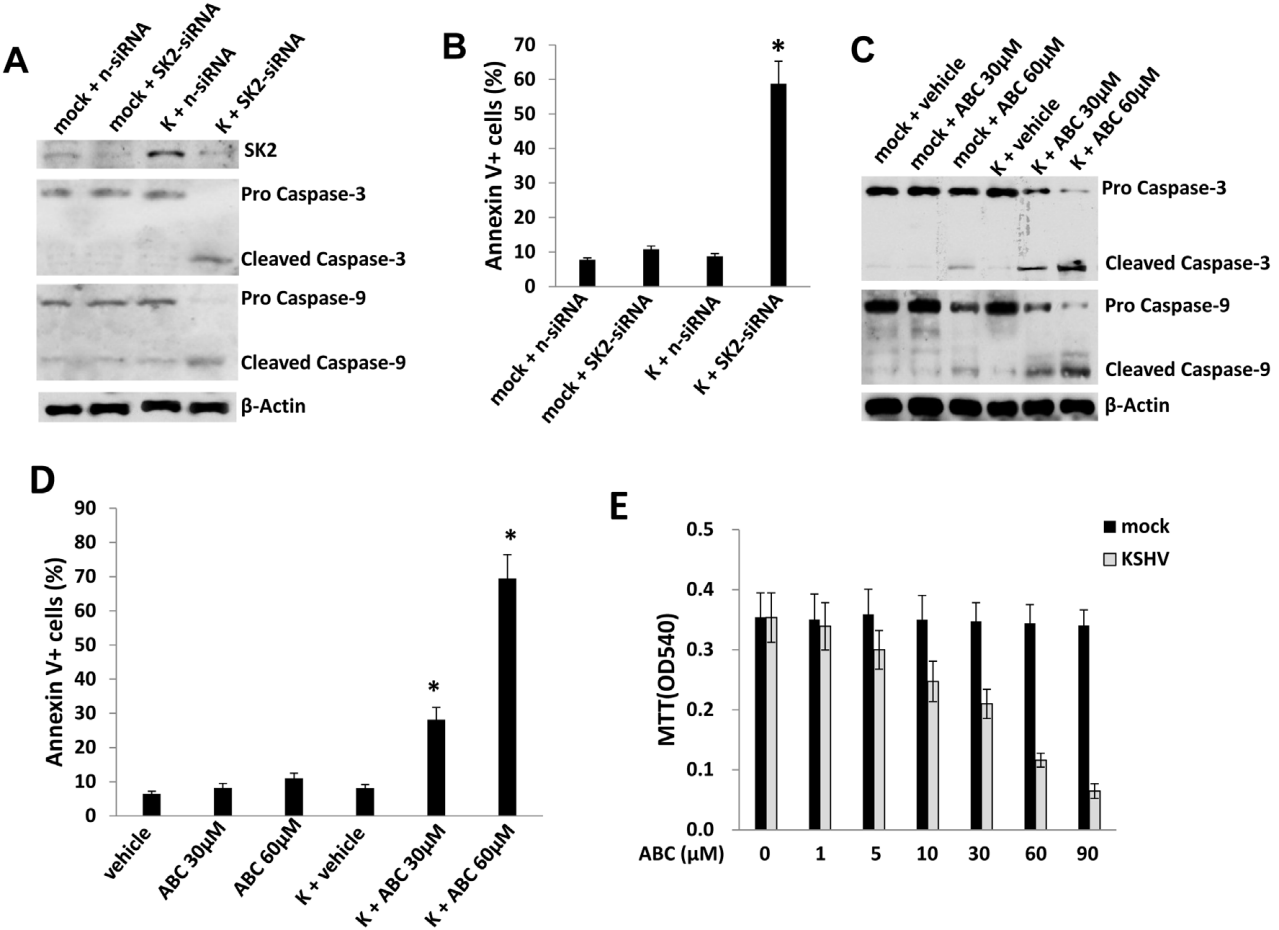
SphK2 maintains survival selectively for KSHV-infected endothelial cells. (**A, B**) pDMVEC were incubated with purified KSHV or control media (mock) for 2 h. 24 h later, cells were transfected with either control non-target (n-siRNA) or SphK2-siRNA (SK2-siRNA) for an additional 48 h. Immunoblots (A) and flow cytometry (B) were used to confirm target knockdown and identify caspase cleavage and apoptosis, respectively, as described in Methods. Error bars represent the S.E.M. for three independent experiments. * = p<0.01 (relative to K+n-siRNA group). (**C–E**) pDMVEC were incubated with or without purified KSHV for 2 h and after 24 h, incubated with the indicated concentrations of ABC294640 (ABC) or vehicle for an additional 24 h. Protein expression, apoptosis, and mitochondrial fitness were quantified using immunoblots, flow cytometry and standard MTT assays, respectively. Error bars represent the S.E.M. for three independent experiments. * = p<0.01 (relative to K+vehicle group).

To determine the impact of targeting SphK2 in the presence or absence of KSHV, we incubated pDMVEC with or without KSHV, then subsequently transfected cells with either SphK2-specific or control siRNA. We found that KSHV increases SphK2 expression in pDMVEC, and that SphK2-specific siRNA reduces SphK2 expression for both control and KSHV-infected pDMVEC, although this effect is more readily demonstrated for infected cells given the relatively low basal expression of SphK2 in uninfected cells (Fig. 1A). SphK2-specific siRNA also induced caspase cleavage and apoptosis for KSHV-infected cells, but had no discernable impact for uninfected pDMVEC (Fig. 1A and B). Consistent with these results, we found that pharmacologic inhibition of SphK2 also induced dose-dependent caspase cleavage and apoptosis for KSHV-infected cells, while only a little increasing of caspase cleavage and apoptosis for ABC294640-treated uninfected cells even at the high concentration of 60 µM used (Fig. 1C–E).

**Figure 2:**
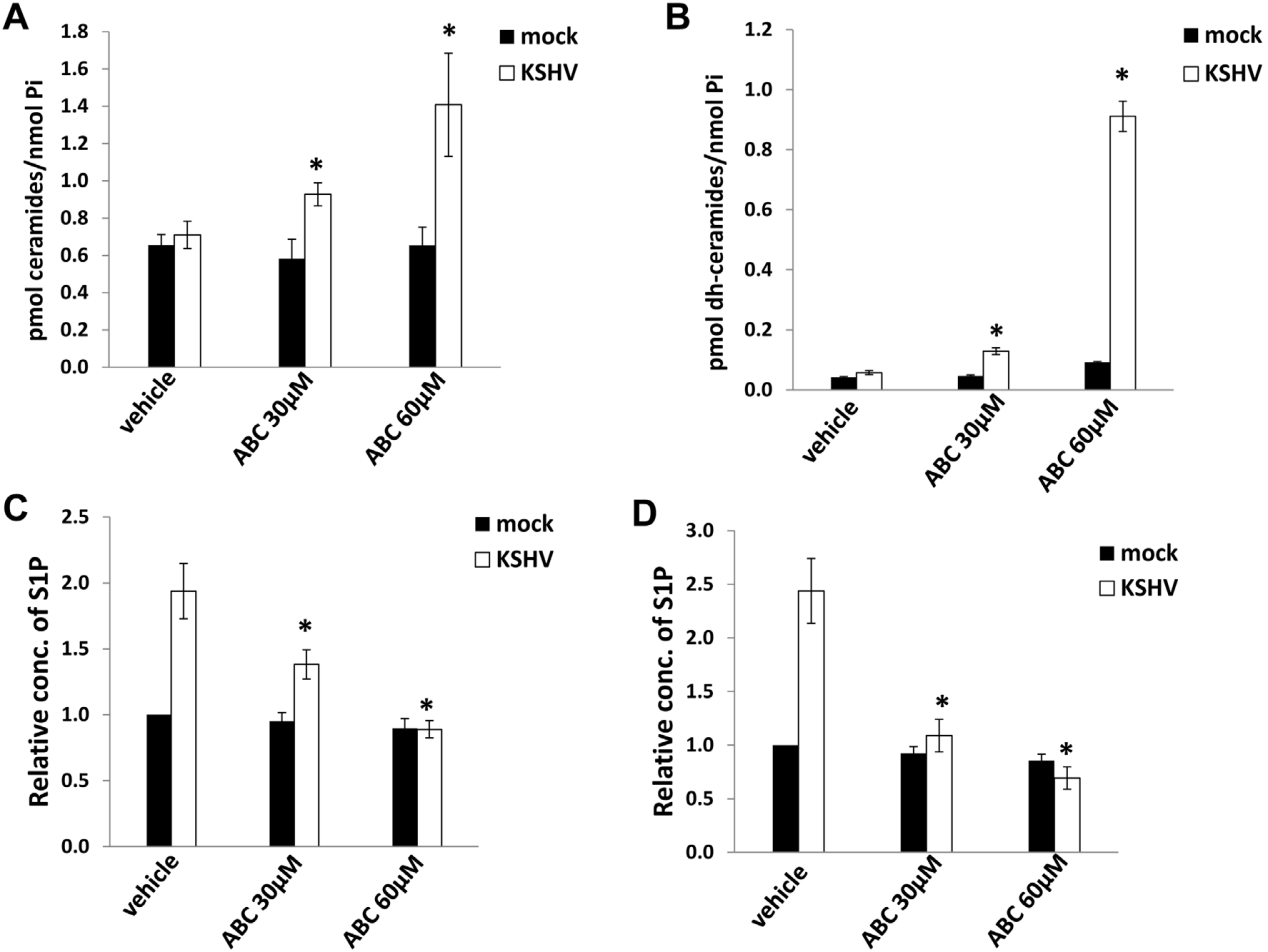
Targeting SphK2 increases accumulation of ceramide species and reduces S1P levels within endothelial cells. (**A, B**) pDMVEC were incubated with or without purified KSHV for 2 h. After an additional 24 h, cells were incubated with the indicated concentrations of ABC294640 (ABC) or vehicle for another 24 h, then ceramide and dihydro-ceramide (dh-ceramide) species quantified as described in Methods. (**C, D**) Cells were treated as (A), then the concentrations of intracellular (C) or extracellular (D) S1P was quantified by ELISA. Error bars represent the S.E.M. for three independent experiments. * = p<0.01.

In parallel, mass spectrometry was used to quantify bioactive sphingolipids and verify functional SphK2 inhibition in pDMVEC. SphK2 inhibition was confirmed within KSHV-infected cells by observation of dose-dependent increases in ceramide and dihydro-ceramide species and reductions in both intracellular and extracellular S1P during pharmacologic inhibition of the enzyme (Fig. 2). In contrast, pharmacologic targeting of SphK2 had no appreciable impact on basal levels of bioactive sphingolipids within uninfected cells (Fig. 2). These data indicate that targeting SphK2 suppresses increased production of S1P induced during KSHV infection of endothelial cells, but not basal production of S1P.

### Inhibition of SphK2 enhances apoptosis through induction of viral lytic gene expression within KSHV-infected endothelial cells

**Figure 3:**
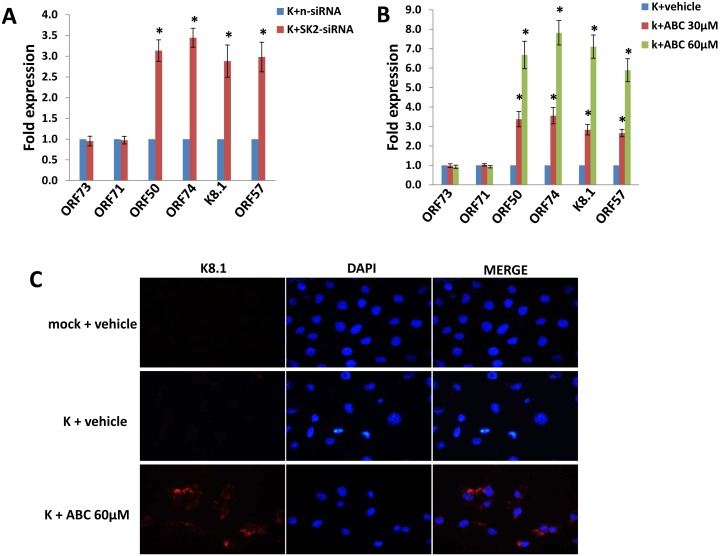
SphK2 suppresses KSHV lytic gene expression within infected endothelial cells. (**A, B**) pDMVEC were incubated with purified KSHV for 2 h, then transfected with either control non-target (n-siRNA) or SphK2-siRNA (SK2-siRNA) for additional 48 h (A), or incubated with the indicated concentrations of ABC294640 (ABC) or vehicle (negative control) for 24 h (B). Viral latent (*ORF73*) and lytic gene (*ORF50*, *ORF74*, *K8.1, ORF57*) transcripts were quantified using qRT-PCR. Error bars represent the S.E.M for three independent experiments. * = p<0.01 (relative to K+n-siRNA or K+vehicle groups). (**C**) Cells were incubated with vehicle or 60 µM ABC for 24 h prior to identification of K8.1 (lytic) protein expression by immunofluorescence.

**Figure 4:**
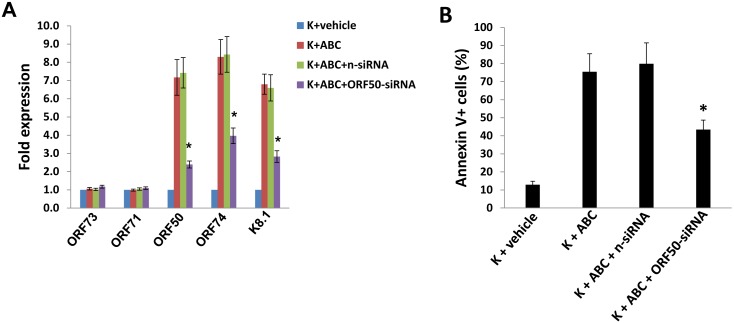
Repression of KSHV lytic gene expression restores viability for KSHV-infected endothelial cells during SphK2 targeting. (A) pDMVEC were incubated with or without purified KSHV for 2 h. After an additional 24 h, cells were transfected with either non-target control siRNA (n-siRNA) or *ORF50*-siRNA for 48 h. Subsequently, cells were incubated with either vehicle or 60 µM ABC for additional 24 h, prior to quantification of KSHV latent (*ORF73* and *ORF71*) and lytic (*ORF50*, *ORF74*, *K8.1*) transcripts using qRT-PCR. (B) In parallel, apoptosis was quantified using flow cytometry. Error bars represent the S.E.M. for three independent experiments. * = p<0.01 (relative to K+ABC+n-siRNA group).

Based on the above results, we hypothesized that SphK2 activity may regulate cell survival pathways specific to KSHV-infected cells, and specifically those associated with viral gene expression. As with other herpesviruses, the life cycle of KSHV involves two phases: a latent phase during which the virus persists as circularized episomes in the nucleus with only a limited number of genes expressed; and a lytic phase during which the viral genome is linearized and more than 80 genes are expressed, ultimately resulting in release of infectious virions and cell death [19]. Induction of KSHV lytic gene expression, dependent on activation of KSHV ORF50 that encodes a viral replication and transcription activator (RTA), results in PEL cell death [6], [20]–[22]. We also previously reported that pharmacologic inhibition of SphK2 in PEL cells induces KSHV lytic gene expression [18]. Therefore, we sought to determine whether SphK2 regulates KSHV gene expression in endothelial cells, and whether targeting SphK2 induces apoptosis through induction of viral lytic gene expression. Using qRT-PCR, we found that either SphK2 silencing, or pharmacologic inhibition of SphK2 in a dose-dependent manner, significantly increased the expression of representative KSHV lytic genes (*ORF50*, *ORF74*, *K8.1, ORF57*) within KSHV-infected pDMVEC (Fig. 3A and B). Immunofluorescence (IFA) data further confirmed that ABC294640 treatment induced viral lytic protein K8.1 expression in the cytoplasma, when compared with the vehicle-treated controls (Fig. 3C). In contrast, targeting SphK2 had no appreciable impact on expression of KSHV *ORF73* which encodes the latency-associated nuclear antigen (LANA) (Fig. 3A and B). Furthermore, RNAi silencing of *ORF50* suppressed “downstream” expression of other KSHV lytic genes such as *ORF74*, *K8.1* and partially inhibited induction of apoptosis for KSHV-infected pDMVEC with SphK2 inhibition (Fig. 4). Together, these data indicate that selectively “killing” KSHV-infected endothelial cells by targeting SphK2 requires increasing viral lytic gene expression within these cells.

### SphK2 regulates expression of KSHV microRNAs promoting viral latency and cell survival

**Figure 5:**
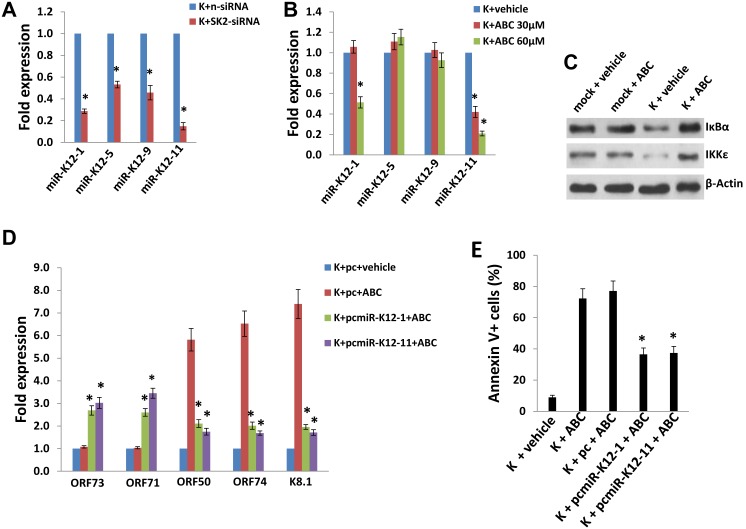
Targeting SphK2 induces KSHV lytic gene expression through suppression of KSHV miRNAs. (**A**) pDMVEC were incubated with purified KSHV or control media (mock) for 2 h. 24 h later, cells were transfected with either control non-target (n-siRNA) or SphK2-siRNA (SK2-siRNA) for an additional 48 h. KSHV miRNA transcripts were quantified using qRT-PCR. (**B**) Cells were incubated with KSHV as in (A) then with the indicated concentrations of ABC294640 (ABC) or vehicle for 24 h prior to qRT-PCR as in (A). Error bars represent the S.E.M for three independent experiments. * = p<0.01 (relative to K+n-siRNA cells or K+vehicle group). (**C**) Cells were prepared as in (A) and incubated with 60 µM ABC for 24 h prior to identification of protein expression using immunoblots. (**D, E**) pDMVEC were incubated with purified KSHV for 2 h then transfected with control vector (pc), or vectors encoding miR-K12-1 (pcmiR-K12-1) or miR-K12-11 (pcmiR-K12-11) for additional 24 h. Thereafter, cells were incubated with vehicle or 60 µM ABC for an additional 24 h. Viral latent (*ORF73, ORF71*) and lytic (*ORF50*, *ORF74*, *K8.1*) transcripts were quantified using qRT-PCR, and apoptosis quantified using flow cytometry. * = p<0.01 (relative to K+pc+ABC group).

KSHV infection induces signal transduction associated with maintenance of viral latency and cell survival, including NF-κB activation [23]–[25]. Moreover, several published studies demonstrate a role for specific KSHV microRNAs (miRNAs) in maintaining viral latency through complimentary mechanisms, including direct targeting of ORF50 or suppression of cellular genes whose products regulate NF-κB activation and other survival pathways [26]–[30]. As noted previously, we reported that PEL cell death was associated with suppression of NF-κB activation during SphK2 inhibition [18], and that apoptosis for KSHV-infected pDMVEC during SphK2 inhibition was mediated in part through activation of ORF50 (Fig. 4). Therefore, we sought to determine whether SphK2 regulates the expression of representative KSHV miRNAs associated with these pathways, included the following: miR-K12-1 which targets IκBα, an inhibitor of NF-κB complexes, thereby promoting NF-κB-dependent viral latency and cell survival [28]; miR-K12-11 which targets IKKε, a signaling intermediate shown previously to facilitate lytic reactivation of KSHV independent of NF-κB activation [30]; miR-K12-5 which targets the Bcl-2-associated factor (BCLAF1), resulting in increased KSHV lytic replication [31]; and miR-K12-9 which targets both BCLAF1 [31] and ORF50 [27], indicating potential competing functions for this miRNA. We found that relative to control siRNA, SphK2-siRNA significantly reduced the expression of miR-K12-1 and miR-K12-11 within KSHV-infected pDMVECs, with a less pronounced effect on the expression of miR-K12-5 and miR-K12-9 (Fig. 5A). Similar results were seen with pharmacologic inhibition of SphK2, including dose-dependent suppression of both miR-K12-1 and miR-K12-11 and no impact on expression of either miR-K12-5 or miR-K12-9 (Fig. 5B). Subsequent experiments revealed that pharmacologic inhibition of SphK2 also restored the expression of known targets of miR-K12-1 and miR-K12-11, IκBα and IKKε, whose expression was reduced with KSHV infection (Fig. 5C). For subsequent “gain-of-function” experiments, we used individual expression constructs encoding either miR-K12-1 or miR-K12-11 to achieve miRNA expression levels approximately 6–8-fold greater than the empty vector control (Fig. S1). We found that this overexpression of either miR-K12-1 or miR-K12-11 repressed KSHV lytic gene expression and apoptosis for KSHV-infected pDMVECs during SphK2 inhibition (Fig. 5D and E).

**Figure 6:**
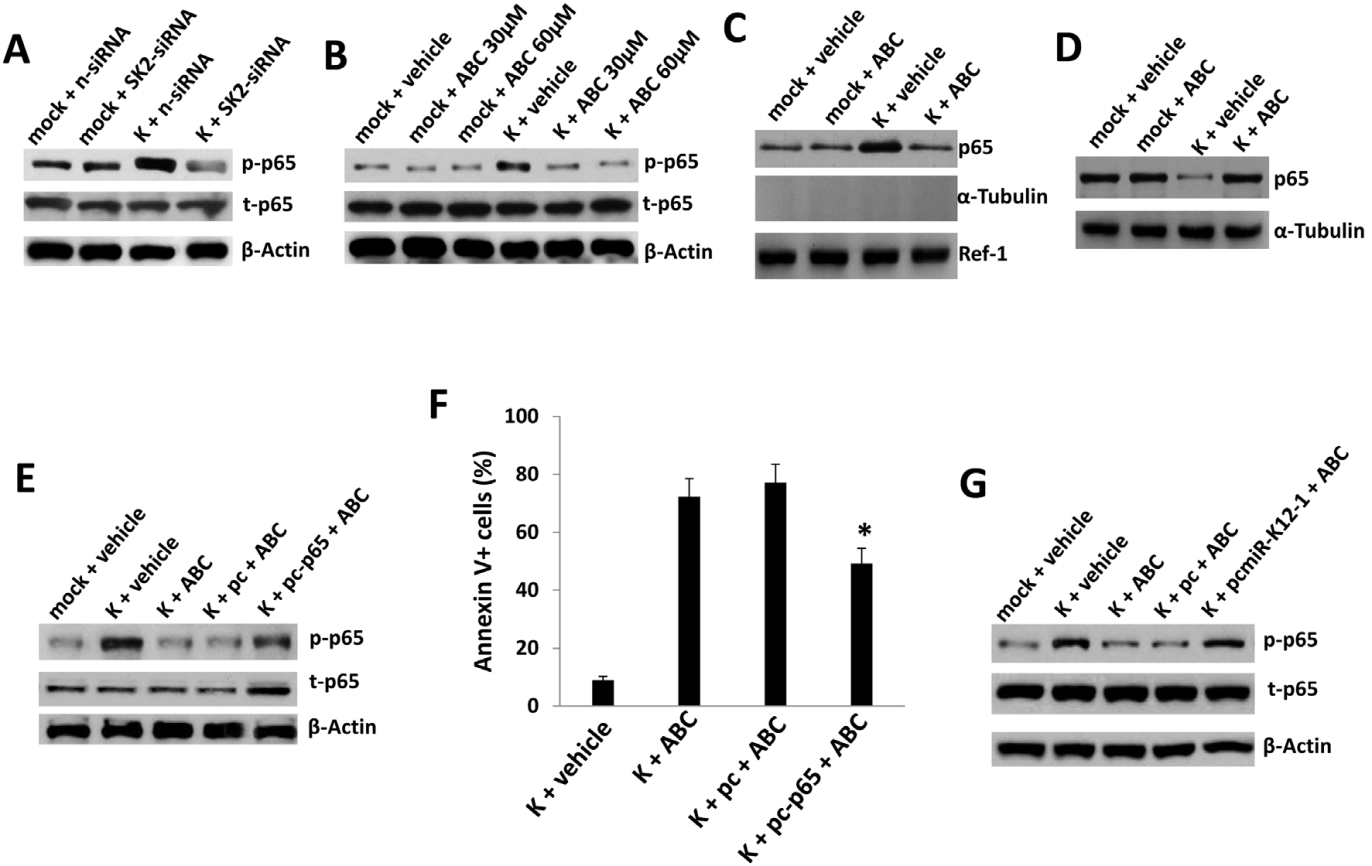
SphK2 supports enhanced NF-κB activation during KSHV infection of endothelial cells. (**A**) pDMVEC were incubated with purified KSHV or control media (mock) for 2 h. 24 h later, cells were transfected with either control non-target (n-siRNA) or SphK2-siRNA (SK2-siRNA) for an additional 48 h. Immunoblots were used to identify protein expression. (**B**) pDMVEC were incubated with KSHV as in (A) and after 24 h, incubated with the indicated concentrations of ABC294640 (ABC) or vehicle for additional 24 h prior to completion of immunoblots. (**C–D**) Cells were treated as (B) prior to nuclear or cytoplasma fractions isolated as described in Methods. Immunoblots were performed to detect signaling molecules, Ref-1 as a positive internal control for nuclear protein expression/loading, and α-Tubulin to exclude the possibility of contamination of nuclear fractions with extranuclear proteins (C). α-Tubulin was used as a loading control for cytoplasma proteins (D). (**E–G**) Cells were incubated with KSHV as in (A) and 24 h later, transfected with control vector (pc), or vectors encoding NF-κB p65 (E and F) or miR-K12-1 (G) for an additional 24 h. Thereafter, cells were incubated with either vehicle or 60 µM ABC for 24 h. Protein expression and apoptosis were determined as previously described. Error bars represent the S.E.M. for three independent experiments. * = p<0.01 (relative to K+pc+ABC group).

miR-K12-1 promotes NF-κB activation in KSHV-infected cells through targeting of IκBα [28]. Therefore, we sought to characterize NF-κB activation during SphK2 targeting. We confirmed that both SphK2-siRNA and pharmacologic inhibition of SphK2 suppressed KSHV-induced phosphorylation of p65, a key constituent of NF-κB complexes (Fig. 6A and B). In addition to p65 phosphorylation, nuclear translocation of p65 is a key step in canonical NF-κB-mediated gene transactivation [47]. Our results confirmed that SphK2 inhibition by ABC294640 significantly reduced KSHV-induced nuclear translocation of p65 but not in the mock cells (Fig. 6C and D). Furthermore, ectopic expression of p65 restored p65 activation and significantly reduced apoptosis for KSHV-infected pDMVECs during SphK2 inhibition (Fig. 6E and F). In support of the selectivity of SphK2 targeting for KSHV-infected cells, neither SphK2-siRNA nor pharmacologic inhibition of SphK2 reduced basal p65 activation within uninfected cells (Fig. 6A and B). Furthermore, we confirmed that miR-K12-1 overexpression restored p65 activation for KSHV-infected pDMVEC during SphK2 inhibition (Fig. 6G).

## Discussion

Pharmacologic inhibition of SphK2 reduces tumor growth in pre-clinical models for breast, prostate, colon, and hepatocellular cancers [17], [31]–[34], as well as KSHV-associated PEL [18]. While cooperative mechanisms for SphK regulation of tumor pathogenesis have been established [7]–[10], data supporting a role for sphingolipid biosynthetic pathways in regulation of viral gene expression and pathogenesis are much more limited in scope [11]–[13]. We have found that targeting SphK2 induces apoptosis selectively for KSHV-infected primary endothelial cells, with minimal for uninfected endothelial cells. Moreover, this occurs in part through activation of KSHV lytic gene expression and suppression of KSHV miRNAs involved in maintaining viral latency and NF-κB activation. We also noted increased the expression of SphK2 following KSHV infection, but additional work is needed to identify the mechanisms associated with this observation and whether SphK2 inhibition results in release of infectious KSHV particles which may justify evaluation of combination therapeutic approaches *in vivo* using ganciclovir or other inhibitors of KSHV replication. Nevertheless, our results are in general agreement with a single study reporting cytomegalovirus (CMV) regulation of lipid biosynthesis pathways wherein CMV infection increased SphK activity, SphK1 and SphK2 mRNA expression, and SphK1 protein expression following *de novo* infection [12]. Although inhibition of SphK1 using either RNA interference or pharmacologic approaches reduced the expression of CMV-encoded genes and CMV replication in the latter study, a time-dependent shift in the effects of CMV infection on *de novo* sphingolipid biosynthesis was noted, including activation within 24 h of infection and inhibition at later time points [12]. We further observed pharmacologic inhibition of SphK2 function within KSHV-infected cells (as demonstrated by dose-dependent accumulation of ceramides and reductions in intracellular and extracellular S1P) but no changes in basal levels of bioactive sphingolipids within uninfected cells with exposure to even high concentrations of ABC294640 exceeding achievable concentrations *in vivo*. It is currently unknown whether this selective effect for KSHV-infected cells is related to increased expression of SphK2, but we feel it is unrelated to penetration or activity of ABC294640 given that multiple studies demonstrate its inhibitory activity for SphK2 within a variety of virus-negative cell types [14]–[17]. Given the selectivity of the drug for SphK2 versus SphK1 [15], our data may also support compensatory or complimentary production of S1P by SphK1 within endothelial cells which has been previously described [35] and which may sufficiently compensate for the loss of S1P production in uninfected cells. S1P binds to 5 G-protein–coupled S1P receptors (S1PR1–5) that activate diverse downstream signaling pathways and facilitate signal transduction following their engagement by S1P in autocrine or paracrine fashion as reviewed elsewhere [36]. Since we find no discernable inhibition of basal activation of p65 or induction of caspase cleavage within uninfected endothelial cells during SphK2 knockdown or pharmacologic targeting of the enzyme, we hypothesize that uninfected cells may be more resistant to killing through this mechanism due to predominant SphK1 activity and “inside-out” signaling for S1P. The latter concept is supported by our data indicating that inhibition of SphK2 reduces both intracellular and extracellular S1P levels in KSHV-infected endothelial cell cultures.

To our knowledge, our study represents the first report indicating a putative role for bioactive sphingolipids in the regulation of viral miRNA expression. Our study is limited in its focus on four representative KSHV miRNAs, although these were chosen based on published data indicating their involvement in regulation of viral latency and NF-κB activation, including either maintenance of latency (miR-K12-1 and miR-K12-11) [28], [30], induction of lytic reactivation (miR-K12-5) [31], or both (miR-K12-9) [27], [31]. A more exhaustive approach may identify additional KSHV miRNAs regulated by SphK2 and for which target genes known to promote viral latency and anti-apoptotic signaling for KSHV-infected cells have been identified [26], [27], [29], [30], [37]–[39]. SphK2 knockdown or pharmacologic targeting resulted in reduced expression or miR-K12-1 and miR-K12-11 with lesser or no impact on miR-K12-5 and miR-K12-9, suggesting that SphK2 and/or S1P may not uniformly regulate expression of all KSHV miRNAs within infected cells. Additional work is required to elucidate mechanisms for SphK2 regulation of KSHV miRNAs, and for regulation of the “latent to lytic” switch more generally, although suppression of lytic reactivation during SphK2 inhibition through ectopic expression of miR-K12-1 and/or miR-K12-11 indicates that SphK2 activity, expression of specific KSHV miRNAs, and lytic reactivation are interdependent. In addition, ectopic expression of individual KSHV miRNA, although achieving expression levels 6–8 fold greater than basal levels, was unable to restore viability to a large proportion of cells during SphK2 inhibition despite restoration of signaling events and lytic gene expression to near pre-treatment levels. It is conceivable, therefore, that S1P impacts KSHV miRNA expression through alternative mechanisms, and that these mechanisms impact other viral or cellular genes mediating cell survival. These issues highlight the need for further clarification of the respective roles of SphK1 and SphK2 in regulating viral gene expression.

Activation of canonical NF-κB activation during KSHV infection promotes viral latency and survival for KSHV-infected cells [40], [41], and previously published data indicate that inhibition of SphK2 suppresses NF-κB activation in tumor cell lines [15], [31]–[33]. We found that SphK2 supports NF-κB activation (p65 phosphorylation and nuclear translocation) in KSHV-infected cells, and that restoration of miR-K12-1 expression restores NF-κB activation and endothelial cell viability during SphK2 targeting. A recent study also provides solid evidence that multiple KSHV miRNAs can target IκBα and regulate the NF-κB pathway activity [42]. Although these data suggest that SphK2 regulation of miR-K12-1 results in NF-κB activation and maintenance of cell survival, they do not exclude a role for other SphK2-regulated signaling pathways in cell survival, including MAPK and Akt pathways which are also regulated by SphK [35], [36] and which were downregulated within PEL cells in dose-dependent fashion with pharmacologic targeting of SphK2 [18]. In fact, SphK1 production of S1P and activation of these alternative pathways through engagement of S1PR or other mechanisms may contribute to resistance of uninfected cells to SphK2 targeting. Furthermore, signaling pathways themselves may regulate viral miRNA expression at the varied steps of miRNA biogenesis or biological modification processing as reviewed elsewhere [43], [44].

Taken together, our data indicate that SphK2 promotes survival selectively for KSHV-infected cells through maintenance of KSHV latency and its regulation of KSHV miRNAs which govern signal transduction and the KSHV lytic switch. This work justifies additional studies to identify mechanisms for SphK2 regulation of KSHV miRNAs. More importantly, they further solidify SphK2 as a potential therapeutic target for KSHV-associated tumors and rationalize clinical trials exploring the utility of ABC294640 for the treatment of KS and PEL.

## Materials and Methods

### Cell culture and reagents

Primary human dermal microvascular endothelial cells (pDMVEC) were purchased from American Type Culture Collection (ATCC) and maintained according to the manufacturer’s instructions as described previously [23]. Human embryonic kidney (HEK) 293A cells were purchased from ATCC and maintained in Dulbecco’s modified Eagle’s medium (DMEM; Gibco) supplemented with 10% FBS, 100 U/ml penicillin, and 100 µg/mL streptomycin. KSHV-infected body cavity-based lymphoma (BCBL-1) cells were purchased from ATCC and maintained in RPMI 1640 media (Gibco) supplemented with 10% fetal bovine serum (FBS), 10 mM HEPES (pH 7.5), 100 U/mL penicillin, 100 µg/mL streptomycin, 2 mM L-glutamine, 0.05 mM β-mercaptoethanol, and 0.02% (wt/vol) sodium bicarbonate [18]. To obtain purified KSHV, BCBL-1 cells were incubated with 0.6 mM valproic acid for 6 days, and purified virus concentrated from culture supernatants and infectious titers determined using pDMVEC as previously described [45]. A MOI of approximately 5 was used for all experiments. 3-(4-chlorophenyl)-adamantane-1-carboxylic acid (pyridin-4-ylmethyl) amide (ABC294640) was synthesized for all experiments as previously described [46].

### Cell viability assays

MTT assays were used for assessment of proliferative capacity as described previously [47]. Flow cytometry was used for quantitative assessment of apoptosis using the FITC-Annexin V/propidium iodide (PI) Apoptosis Detection Kit I (BD Pharmingen) according to the manufacturer’s instructions.

### Transfection Assays

pDMVEC were transfected using pcDNA3.1-FLAG-NF-κB p65 (pc-p65) or control vectors as described previously [48]. Cells were also transfected in 12-well plates using Lipofectamine 2000 (Invitrogen) for 24 to 48 h and constructs for overexpression of miR-K12-1 and miR-K12-11 as previously described [49]. For RNA interference, pDMVEC were transfected for 48 h with either SK2-, ORF50-, or control (non-target)-siRNAs using ON-TARGET plus SMART pool (Dharmacon) and DharmaFECT Transfection Reagent (Dharmacon) according to the manufacturer’s instructions. Transfection efficiency was normalized through co-transfection of a lacZ reporter construct and determination of β-galactosidase activity using a commercial β-galactosidase enzyme assay system according to the manufacturer’s instructions (Promega). For some experiments, transfection efficiency was further assessed on a single-cell level through co-transfection with a green fluorescent protein (GFP) construct (pEGFP-N1) and subsequent flow cytometry analysis as described previously [50]. This procedure was performed using pDMVEC and HEK293A cells (as a positive control), since the transfection efficiency of GFP for HEK293A cells approaches 90%. Using this method, we confirmed the pDMVEC transfection efficiency of approximately 50% of cells in our experiments (Fig. S2).

### Immunoblotting

Cells were lysed in buffer containing 20 mM Tris (pH 7.5), 150 mM NaCl, 1% NP40, 1 mM EDTA, 5 mM NaF and 5 mM Na_3_VO_4_. Total cell lysates (30 µg) were resolved by 10% SDS–PAGE, transferred to nitrocellulose membranes, and incubated with 100–200 µg/mL antibodies as follows: phospho-NF-κB p65 (Ser536), total NF-κB p65, pro−/cleaved caspase-3, pro-/cleaved caspase-9, IκBα, IKKε, Ref-1 (Cell Signaling Technologies) and α-Tubulin (Sigma). For loading controls, lysates were also incubated with antibodies detecting β-Actin (Sigma). Immunoreactive bands were developed using an enhanced chemiluminescence reaction (Perkin-Elmer). Nuclear and cytoplasma protein fractions were isolated using a Nuclear Extract Kit (Active Motif) as previously described [48], and the nuclear origin of these extracts was verified using anti-Ref-1 antibodies. Anti-α-Tubulin antibodies were used to exclude contamination of nuclear extracts with extranuclear proteins.

### qRT-PCR

Total RNA was isolated using the RNeasy Mini kit according to the manufacturer’s instructions (QIAGEN). cDNA was synthesized from equivalent total RNA using SuperScript III First-Strand Synthesis SuperMix Kit (Invitrogen) also according to the manufacturer’s procedures. Primers used for amplification of target genes are displayed in Table S1. Amplification was carried out using an iCycler IQ Real-Time PCR Detection System, and cycle threshold (Ct) values were tabulated in duplicate for each gene of interest in each experiment. “No template” (water) controls were used to ensure minimal background contamination. Using mean Ct values tabulated for each gene, and paired Ct values for β-actin as an internal control, fold changes for experimental groups relative to assigned controls were calculated using automated iQ5 2.0 software (Bio-rad). For amplification of viral miRNAs, cDNA was synthesized using the Taqman miRNA RT kit (Applied Biosystems), and qPCR was performed using the Taqman MicroRNA Assays kit (Applied Biosystems) and a 7500 Real Time PCR System. Fold changes for microRNA were calculated using paired Ct values for RNU6B as recommended by the manufacturer (Applied Biosystems).

### Immunofluorescence

1×10^4^ pDMVEC per well were seeded in eight-well chamber slides (Nunc) and incubated with dilutions of freshly prepared viral stocks in the presence of 8 µg/mL Polybrene (Sigma-Aldrich) for 2 h at 37°C. After remaining in culture overnight, cells were treated with indicated concentration of ABC294640 for 24 h. cells were incubated in 1∶1 methanol-acetone at −20°C for fixation and permeabilization, then with a blocking reagent (10% normal goat serum, 3% bovine serum albumin, and 1% glycine) for an additional 30 minutes. Cells were then incubated for 1 h at 25°C with 1∶2000 dilution of a mouse anti-K8.1 monoclonal antibody (ABI) followed by 1∶200 dilution of a goat anti-mouse secondary antibody conjugated to Texas Red (Invitrogen). For identification of nuclei, cells were subsequently counterstained with 0.5 µg/mL 4′,6-diamidino-2-phenylindole (DAPI; Sigma) in 180 mM Tris-HCl (pH 7.5). Slides were washed once in 180 mM Tris-HCl for 15 minutes and prepared for visualization using a Leica TCPS SP5 AOBS confocal microscope.

### Sphingolipid analyses

Quantification of ceramide and dihydro-ceramide species was performed using a Thermo Finnigan TSQ 7000 triple-stage quadruple mass spectrometer operating in Multiple Reaction Monitoring positive ionization mode (Thermo Fisher Scientific). Quantification was based on calibration curves generated by spiking an artificial matrix with known amounts of target standards and an equal amount of the internal standard. The target analyte: internal standard peak area ratios from each sample were compared with the calibration curves using linear regression. Final results were expressed as the ratio of sphingolipid normalized to total phospholipid phosphate level using the Bligh and Dyer lipid extract method [51].

### Quantification of S1P

Concentrations of S1P in culture supernatants and pDMVEC cell lysates were determined using the S1P Assay Kit (Echelon) according to the manufacturers’ instructions.

### Statistical analysis

Significance for differences between experimental and control groups were determined using the two-tailed Student’s t-test (Excel 8.0) and p values<0.01 were considered significant.

## Supporting Information

Figure S1 **Restoration of KSHV miRNA expression during SphK2 targeting.** pDMVEC were incubated with KSHV for 2 h. 24 h later, cells were transfected with control vector (pc), or vectors encoding either miR-K12-1 or miR-K12-11 for an additional 24 h prior to their incubation with either vehicle or 60 µM ABC for another 24 h. miRNA expression was determined as previously described. Error bars represent the S.E.M. for three independent experiments.
(TIF)

Figure S2 **The transfection efficiency within pDMVEC.** pDMVEC and HEK293A cells (as a positive control) were transfected with or without pEGFP-N1 vector by Lipofectamine 2000 (Invitrogen) for 24 h, then transfection efficiency was assessed by flow cytometry.
(TIF)

Table S1 **Primer sequences for qRT-PCR in this study.**
(DOCX)
