# Supplementary figures and images for: Sphingosine Kinase-2 Maintains Viral Latency and Survival for KSHV-Infected Endothelial Cells
Source: PLoS One. 2014 Jul 10;9(7):e102314. doi: 10.1371/journal.pone.0102314 (PMC4092155; doi:10.1371/journal.pone.0102314)

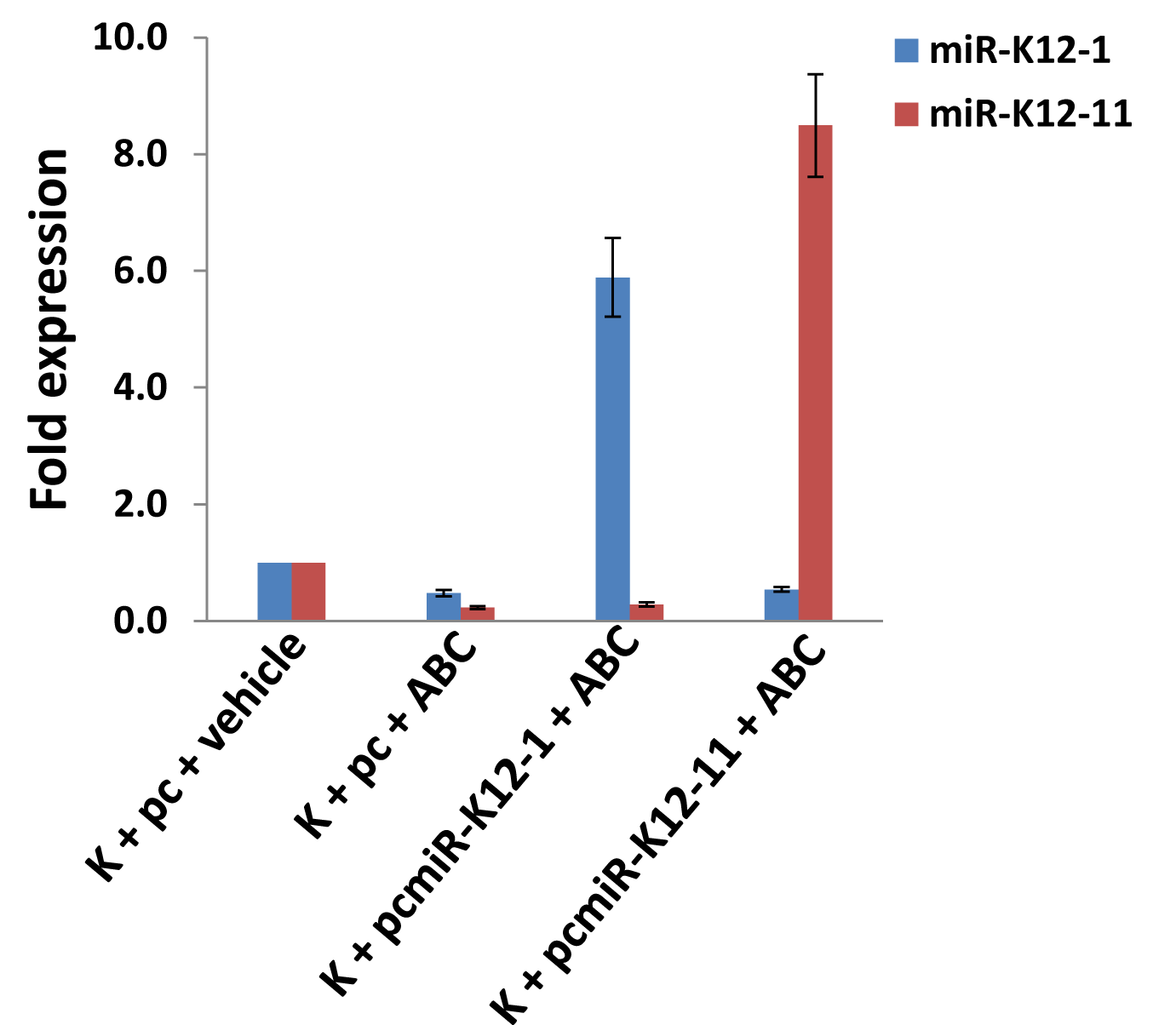

Supplement: Figure S1 — Restoration of KSHV miRNA expression during SphK2 targeting. pDMVEC were incubated with KSHV for 2 h. 24 h later, cells were transfected with control vector (pc), or vectors encoding either miR-K12-1 or miR-K12-11 for an additional 24 h prior to their incubation with either vehicle or 60 µM ABC for another 24 h. miRNA expression was determined as previously described. Error bars represent the S.E.M. for three independent experiments. (TIF) [file pone.0102314.s001.tif]

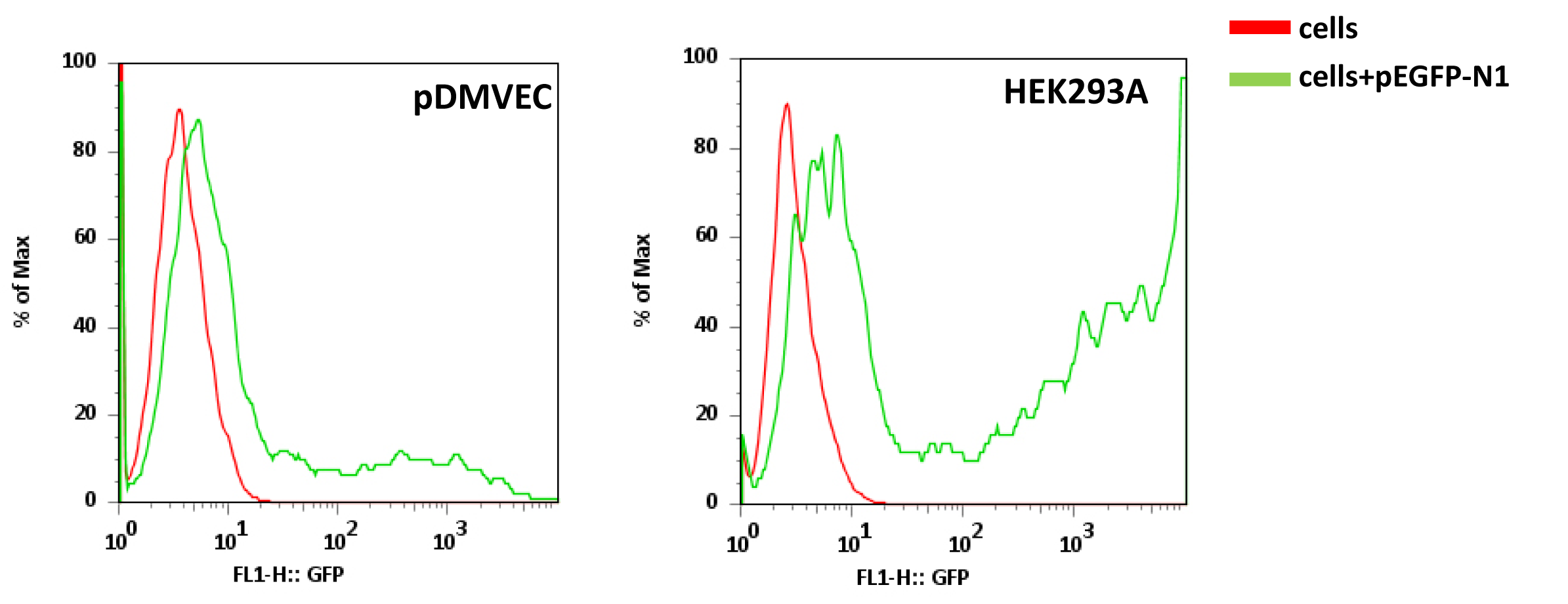

Supplement: Figure S2 — The transfection efficiency within pDMVEC. pDMVEC and HEK293A cells (as a positive control) were transfected with or without pEGFP-N1 vector by Lipofectamine 2000 (Invitrogen) for 24 h, then transfection efficiency was assessed by flow cytometry. (TIF) [file pone.0102314.s002.tif]
