# Supplementary material for: Sphingosine Kinase-2 Maintains Viral Latency and Survival for KSHV-Infected Endothelial Cells
Source: PLoS One. 2014 Jul 10;9(7):e102314. doi: 10.1371/journal.pone.0102314 (PMC4092155; doi:10.1371/journal.pone.0102314)
Supplement: Table S1 — Primer sequences for qRT-PCR in this study. (DOCX) [file pone.0102314.s003.docx]

**Table S1. Primer sequences for qRT-PCR.**

| **Gene** | **Sequences (5’ 3’)** |
| --- | --- |
| *ORF73 (Lana)* | *sense TCCCTCTACACTAAACCCAATA*  *antisense TTGCTAATCTCGTTGTCCC* |
| *ORF71 (vFlip)* | *sense GGGCACGGATGACAGGGAA*  *antisense TGTGATGGGCCGGAAAGG* |
| *ORF50 (Rta)* | *sense TAATGTCAGCGTCCACTCC*  *antisense TTCTGGCACGGTCAAAGC* |
| *ORF74 (vGpcr)* | *sense CATCCGCTGCACTGTTAA*  *antisense GCTTTGTCCTCCTCACCA* |
| *K8.1* | *sense CACCACAGAACTGACCGATG*  *antisense TGGCACACGGTTACTAGCAC* |
| *ORF57* | *sense GGGTGGTTTGATGAGAAGGA*  *antisense CGCTACCAAATATGCCACCT* |
| *β-actin* | *sense GGAAATCGTGCGTGACATT*  *antisense GACTCGTCATACTCCTGCTTG* |
